# A Review on Affordable Combinations in Type 2 Diabetes Care: Exploring the Cost-Effective Potential of Glipizide + Metformin and Glimepiride + Metformin + Pioglitazone

**DOI:** 10.7759/cureus.59850

**Published:** 2024-05-07

**Authors:** Shehla Shaikh, Vishal Vaidya, Amit Gupta, Raghunath Kulkarni, Ashok Joshi, Medhinee Kulkarni, Vidhe Sharma, Santosh Revankar

**Affiliations:** 1 Endocrinology, Saifee Hospital, Mumbai, IND; 2 Endocrinology, Sir H. N. Reliance Foundation Hospital, Mumbai, IND; 3 Diabetes and Endocrinology, Diacare Clinic, Ahmedabad, IND; 4 Diabetes and Endocrinology, Centre for Diabetes Care, Greater Noida, IND; 5 Diabetes and Endocrinology, Sevasadhan Superspeciality Centre, Sangli, IND; 6 Endocrinology and Diabetes, Balaji Hospital, Thane, IND; 7 Diabetes and Endocrinology, Lifespan Diabetes and Cardiometabolic Clinic, Mumbai, IND; 8 Diabetes and Endocrinology, Ruby Hall Clinic Hinjawadi, Pune, IND; 9 Scientific Services, USV Private Limited, Mumbai, IND

**Keywords:** sustainable healthcare, affordability, glycemic control, cost-effective, glimepiride with metformin and pioglitazone, glipizide and metformin, combination drugs, diabetes mellitus

## Abstract

Management of type 2 diabetes mellitus (T2DM) largely relies on medication adherence of individuals with diabetes to achieve optimal glycemic control. The economic burden of diabetes could impede adherence, leading to a reduction in treatment efficacy and increased risk of complications. Furthermore, monotherapy in diabetes is losing traction due to its ineffectiveness in achieving early and sustained optimal glycemic control in a significant proportion of the population. Hence, clinicians prefer combination treatment due to their improved efficacy and safety. Considering these factors, the current review highlights the safety and efficacy of the affordable combination therapies, a dual therapy, glipizide + metformin, and a triple-drug combination of glimepiride + metformin + pioglitazone and its applicability in the management of T2DM among individuals with diabetes in India.

## Introduction and background

Type 2 diabetes mellitus (T2DM), a chronic metabolic disorder, affects individuals across all socioeconomic strata [1]. However, almost 80% of individuals with T2DM are estimated to be residing in lower- and middle-income countries [2]. India, shouldering a significant proportion (101 million) of individuals with diabetes, is also home to a wide sociodemographic profile [3]. There is increasing adoption of the principle of democracy in diabetes to emphasize the importance of accessibility, affordability, and awareness for better reach of diabetes care practices to even the lowest strata of the socioeconomic groups [4]. Participants from the study by Medi et al. [5] in 2015 noted that 56% of patients were non-compliant with their diabetes medication due to financial difficulties. Studies conducted by Divya and Nadig [6] and Manobharathi et al. [7] revealed a significant association between economic constraints and poor medication compliance among diabetes patients, leading to treatment ineffectiveness or failure.

Combination therapy plays a crucial role in reaching glycemic goals in T2DM. When employed judiciously, fixed-dose combinations (FDCs) present a multitude of advantages, including reduced pill load, minimized potential for adverse effects, and enhanced patient adherence, all of which collectively enhance treatment effectiveness [8]. Combination therapies reduce the co-payment burden by consolidating multiple medications into a single pill. Studies show that combination therapies do not increase healthcare costs compared to monotherapy options. This financial relief eases the economic strain on individuals managing chronic conditions such as diabetes [9]. Ultimately, combination therapies streamline treatment, improve patient compliance, and contribute to better long-term health outcomes for diabetic individuals.

## Review

Poor medication adherence and its consequences

Unsatisfactory compliance with antidiabetic medications has been observed in T2DM patients globally [10]. In India, studies with varying criteria for medication adherence have estimated medication non-compliance among diabetes patients to range from 20.5% to 66% [5,11-14]. Lower socioeconomic status, increasing age and number of drugs, and fear of side effects are considered major barriers to effective medication adherence [5].

Non-compliance to diabetes medication is one of the major factors associated with poor glycemic control, increased risk of complications, and cardiorenal events [15,16]. With good adherence to diabetes medication (medication possession ratio ≥ 0.8), a significant reduction in microvascular and macrovascular complications (adjusted hazard ratio (AHR) = 0.96, p = 0.05) is observed [17]. Cardiovascular comorbidities are common among T2DM patients and contribute to morbidity and mortality [16]. Poor adherence (proportion of days covered (PDC) < 80%) to diabetes medication and glycemic control (hemoglobin A1c (HbA1c) > 9%) was associated with a higher risk of cerebrovascular events (HR: 1.68 (95% confidence interval (CI): 1.10-2.57)), cardiovascular disease (HR: 2.15 (95% CI: 1.52-3.04)), and heart failure (HR: 2.02 (95% CI: 1.12-3.63)). In the study by Gatwood et al. [15], the results suggested a significantly higher risk of ischemic stroke (HR: 1.22 (95% CI: 1.05-1.42), p = 0.011) and myocardial infarction (HR: 1.14 (95% CI: 1.03-1.27), p = 0.015) with poor medication adherence (proportion of days covered < 80%).

Another major macrovascular complication in diabetes is chronic kidney disease. A lower risk of renal events (HR: 0.45 (95% CI: 0.32-0.64), p < 0.001) is observed with higher adherence (PDC > 80%) to antihyperglycemic medication [18]. Kuo et al. [19], in a seven-year follow-up study among 908 participants on oral antihyperglycemic medication, noted significantly higher self-reported kidney problems (AHR: 1.59 (95% CI: 1.13-2.23), p = 0.008) in individuals who either discontinued medication, had not been medicated for two weeks, or failed to properly stock their medications. Furthermore, with poor medication adherence (proportion of days covered < 80%), Yaguchi et al. [16] concluded a significantly higher need for dialysis. Taken together, these studies highlight the importance of medication adherence in effective glycemic control and in reducing the morbidity and mortality associated with diabetes complications.

Importance of pharmacoeconomic profile in T2DM

The pharmacoeconomic profile of a drug is an important factor, especially in widely prevalent chronic metabolic conditions such as diabetes. At an individual level, the economic burden, in addition to safety, efficacy, and ease of administration, is a major factor associated with a lack of medication adherence [20]. This trait is observed in a significant proportion (36.9%-75.1%) of T2DM patients [10,21,22] and could hasten the onset of diabetic complications. The direct costs of T2DM management include medical and non-medical expenses borne by individuals and families, while the indirect costs include loss of productivity, among others [23].

In India, with 77 million population diagnosed with T2DM, the economic consideration is more pronounced [24]. The net expenditure on healthcare in India was estimated to be around 31 billion dollars as of 2017 [25].

The average annual household expenditure for diabetes management ranges from ~INR11,500 to ~INR49,000 [26-28], which was seen to exhaust between 5% and 25% or more of a household income [25,28,29]. This indicates that lower-income groups bear a larger proportion of their income for diabetes care. Medication cost was the major expenditure accounting for about 60% [30], which amounts to about INR6,900 to INR29,400.

Furthermore, suboptimal glycemic control can also result in diabetes-associated complications, which have been observed to increase expenses by 2.3- to 3.3-fold [31,32]. In fact, the most expensive aspect of diabetes management involves treating diabetes-mediated complications. In India, the lack of effective use of health insurance and medical reimbursement is suggested to contribute to the out-of-pocket expense incurred in diabetes management [31]. Cost-effective options amicable to the individuals for early effective glycemic control are much desired.

Approaches for the management of T2DM

Traditionally, T2DM management adopts a stepwise approach with lifestyle modification, followed by monotherapy, and an optional combination therapy [33]. However, studies including the UKPDS in 1999 have observed that the proportion of T2DM patients with optimum glycated hemoglobin A1c (HbA1c) levels (<7%) on monotherapy has been declining every year [34], with failure rates of up to 45% [35,36]. The diverse phenotypes of T2DM in India are expected to contribute to the high rates of treatment failure [37]. Polypharmacy has also not yielded benefits in glycemic control or cost [38,39]. While multidrug therapy has been associated with poor compliance, combination therapies have emerged as the preferred option by clinicians [40,41]. Various organizations including the American Diabetes Association and the Research Society for the Study of Diabetes in India suggest combination therapy of metformin with other oral antidiabetic drugs (OADs) in patients with uncontrolled T2DM for over three months on any traditional approach [42,43]. In addition to improving the glycemic profile, the low-dose combinations of OADs also reduce the risk of side effects, improving the safety and efficacy of management [33]. Hence, combination therapy offering multiple mechanisms has emerged as an effective solution for glycemic management and aided in improving medication adherence and reducing the risk of adverse events [44]. By leveraging the improvement in glycemic control, the incidence and the costs associated with diabetes complications are significantly reduced in the long term [45].

Sulfonylureas (SUs) are one of the cost-effective and readily available antiglycemic agents (AGAs). They can be commonly used in combination with metformin and/or thiazolidinediones and can help in reducing costs while improving patient adherence [46]. When compared to monotherapy, previous studies have highlighted the cost-effectiveness of metformin-SU combination therapy [46] and demonstrated efficacy in improving glycemic control (by 0.5% HbA1c reduction) [47]. Similarly, thiazolidinediones such as pioglitazone have also demonstrated effectiveness in diabetes management while conferring cost-effectiveness in combination with other AGAs [48,49]. The current review highlights the cost-effectiveness, safety, and efficacy of two such combination therapies (glipizide + metformin (Glz + Met) and glimepiride + metformin + pioglitazone (Gpd + Met + Pio)) with an added benefit of cost-effectiveness for improving the glycemic profile of diabetes patients in India.

Glipizide + metformin: An affordable solution

Glipizide is a second-generation sulfonylurea approved by the Food and Drug Administration (FDA) in 1994. It is often administered with several oral antiglycemic agents to render maximum benefit. Metformin, a biguanide, reduces liver glucose production and enhances tissue sensitivity to insulin, contributing to overall blood sugar control, and is used in the management of T2DM [50]. Glipizide, in combination with metformin, is effective in achieving effective glycemic control, especially among patients with aberrant glycemic variables over three months despite metformin monotherapy. A multicenter, double-blind, placebo-controlled trial involving 122 patients demonstrated that the addition of 2.5 mg glipizide gastrointestinal therapeutic system (GITS) to existing metformin treatment significantly improved plasma glucose and HbA1c levels over 16 weeks compared to the placebo group [51]. Moreover, a higher percentage of patients in the glipizide GITS group achieved target HbA1c levels of both <7% and <6.5% compared to the placebo group. Glipizide + metformin (5/500 mg) combination therapy achieved a fourfold increase in achieving target HbA1c levels when compared with monotherapy of glipizide (30 mg) and metformin (500 mg) [52]. Glipizide + metformin therapy is well-tolerated as evidenced by improvement in the glycemic index without weight gain and lower incidence of hypoglycemia episodes [51,52]. Glipizide is especially desirable and is considered one of the primary choices by physicians for T2DM management due to its low cost and availability [53]. The cost analysis indicated that Glz + Met is priced at INR2 per tablet. At twice daily administration, the annual cost accounts for INR1,460. This is about 5-20 times lower than the estimated annual cost for medication expenditures [26-28]. The affordability and availability of Glz + Met in India enhance accessibility to the population in need of the medication. The lower cost could also contribute to improved medication adherence, reducing the risk of complications and achieving positive health outcomes for individuals with diabetes in India.

Among SUs, glipizide has the fastest absorption and onset of action and the shortest half-life [54]. The molecular mechanisms of glipizide involve a partial block of the potassium channels in the beta cells of the pancreatic islets. This leads to cellular depolarization and the opening of the voltage-gated calcium channels, stimulating the release of insulin from the pancreatic beta cells. The extra-pancreatic effects shown to be important in the action of glipizide are an increase in insulin sensitivity at peripheral target sites such as muscle, fat, or liver cells and a decrease in hepatic glucose production [55]. Glipizide is typically prescribed at a daily dosage of 2.5-10 mg [56]. Following a single oral dose, peak plasma concentrations are observed within 1-3 hours, and the elimination half-life ranges from two to four hours in normal subjects, whether administered intravenously or orally. Hepatic transformation is the primary route of metabolism for glipizide. Approximately 10% of the administered dose is excreted unchanged in urine and feces [56]. Although glipizide is considered for use in patients with kidney disease due to its hepatic metabolization [56,57], studies on the efficacy of its use in patients with renal disease are warranted.

Glimepiride + metformin + pioglitazone: A budget-friendly alternative

A triple dose combination of Gpd + Met + Pio has demonstrated effective glycemic control, indicated by a decrease of HbA1c levels by 1.3% [58]. Another multicenter study conducted in India increased the achievement of glycemic goals and lipid levels without any severe adverse events [59]. Fixed-dose combinations of glimepiride + metformin have been effective in managing T2DM among newly diagnosed and long-standing diabetes patients [60] in India. Kim et al. [61] noted significant improvement in the fasting plasma glucose and HbA1c levels with FDC of glimepiride metformin among T2DM patients inadequately managed by low-dose metformin monotherapy. The number of patients with effective glycemic control (<7%) was also significantly higher at the end of the study and was well-tolerated. The FDC aids in glycemic control by addressing complementary mechanisms of T2DM, with metformin improving insulin resistance in tissues and glimepiride stimulating insulin secretion from the pancreas [61]. This FDC, especially those with sustained release properties, can also improve medication adherence [62]. Another combination utilized in T2DM management is pioglitazone + glimepiride, which is considered a promising therapeutic option as they together address the pathological defects of T2DM. Glimepiride stimulates insulin release and aids in glucose uptake, while pioglitazone improves insulin sensitivity in peripheral tissues, such as muscles and adipose tissue [63,64]. In addition to the dual glycemic benefits, the pioglitazone + glimepiride combination could provide extra-glycemic benefits in lipid metabolism and improved tolerability in T2DM patients with acute myocardial infarction [40]. Due to their complementary effects on both glycemic and extra-glycemic variables, fixed-dose administration of these components appears a reasonable choice for effective and sustained metabolic control, especially among patients at high risk for developing complications [40]. A triple dose combination of Gpd + Met + Pio is priced at INR6.52 per tablet, with a recommended dosing of once a day. With these dosing recommendations, the annual cost amounts to INR2,380. The cost estimate of this combination is 4-16 times lower than the annual estimates of medication cost in diabetes care [26-28]. Such affordable and easily accessible drug combinations in India could improve adherence, reduce the risk of complications, and contribute to the overall improvement of the quality of life in T2DM patients.

## Conclusions

Efficient T2DM management relies on cost-effective interventions to improve patient adherence. Affordable combination therapies such as Glz + Met and Gpd + Met + Pio offer significant advantages, reducing medication costs by up to 20 times. While their safety and efficacy are well-established, further research is needed to confirm real-world effectiveness and ensure rational use.
